# Comparison of rider stability in a flapless saddle versus a conventional saddle

**DOI:** 10.1371/journal.pone.0196960

**Published:** 2018-06-06

**Authors:** Hilary M. Clayton, Alexandra Hampson, Peter Fraser, Arlene White, Agneta Egenvall

**Affiliations:** 1 Department of Large Animal Clinical Sciences, Michigan State University, East Lansing, Michigan, United States of America; 2 Happy Athlete Sport Therapy, Yellowknife, Northwest Territories, Canada; 3 Pete Fraser Consulting, Oakland, California, United States of America; 4 Animal Rehab Institute, Loxahatchee, Florida, United States of America; 5 Department of Clinical Sciences, Faculty of Veterinary Medicine and Animal Sciences, Swedish University of Agricultural Sciences, Uppsala, Sweden; Universidade do Porto Instituto de Biologia Molecular e Celular, PORTUGAL

## Abstract

The purpose of a saddle is to improve the rider’s safety, security, and comfort, while distributing the forces exerted by the rider and saddle over a large area of the horse’s back without focal pressure points. This study investigates the effects on rider stability of an innovative saddle design that differs from a conventional saddle in having no flaps. Five horses were ridden by their regular rider in their usual saddle and in a flapless saddle. A pressure mat (60 Hz) placed between the saddle and the horse’s back was used to determine the position of the center of pressure, which represents the centroid of pressure distribution on the horse’s back. Data were recorded as five horses were ridden at collected and extended walk, trot and canter in a straight line. Data strings were split into strides with 5 strides analysed per horse/gait/type. For each stride the path of the rider’s center of pressure was plotted, maximal and minimal values in the anteroposterior and mediolateral directions were extracted, and ranges of motion in anteroposterior and mediolateral directions were calculated. Differences between the conventional and flapless saddles were analysed using mixed models ANOVA. Speed and stride length of each gait did not differ between saddles. Compared with the conventional saddle, the flapless saddle was associated with significant reductions in range of motion of the rider’s center of pressure in the mediolateral direction in all gaits and in the anteroposterior direction in collected trot, extended trot and extended canter. The improved stability was thought to result from the absence of saddle flaps allowing the rider’s thighs to lie in more adducted positions, which facilitated the action of the lumbopelvic-hip musculature in stabilizing and controlling translations and rotations of the pelvis and trunk. The closer contact between rider and horse may also have augmented the transfer of haptic information.

## Introduction

For at least 4,000 years horses have been ridden by people as a means of transportation, as an instrument of warfare and as a companion in sport [1]. In developed countries, horses have become less utilitarian over the past 50 years and are now primarily used for sport and recreation. Saddles have been developed to improve the comfort and security of the rider and, over the years, have evolved according to the different purposes for which horses have been used. As sporting performances have improved, trainers have realized that both horse and rider perform better when the equipment they use is optimally designed and fitted. As a result, there have been many innovations in the design of bits, bridles, nosebands and saddles with the objectives of improving comfort, performance or safety. However, relatively few have been subjected to rigorous scientific evaluation.

**Fig 1:**
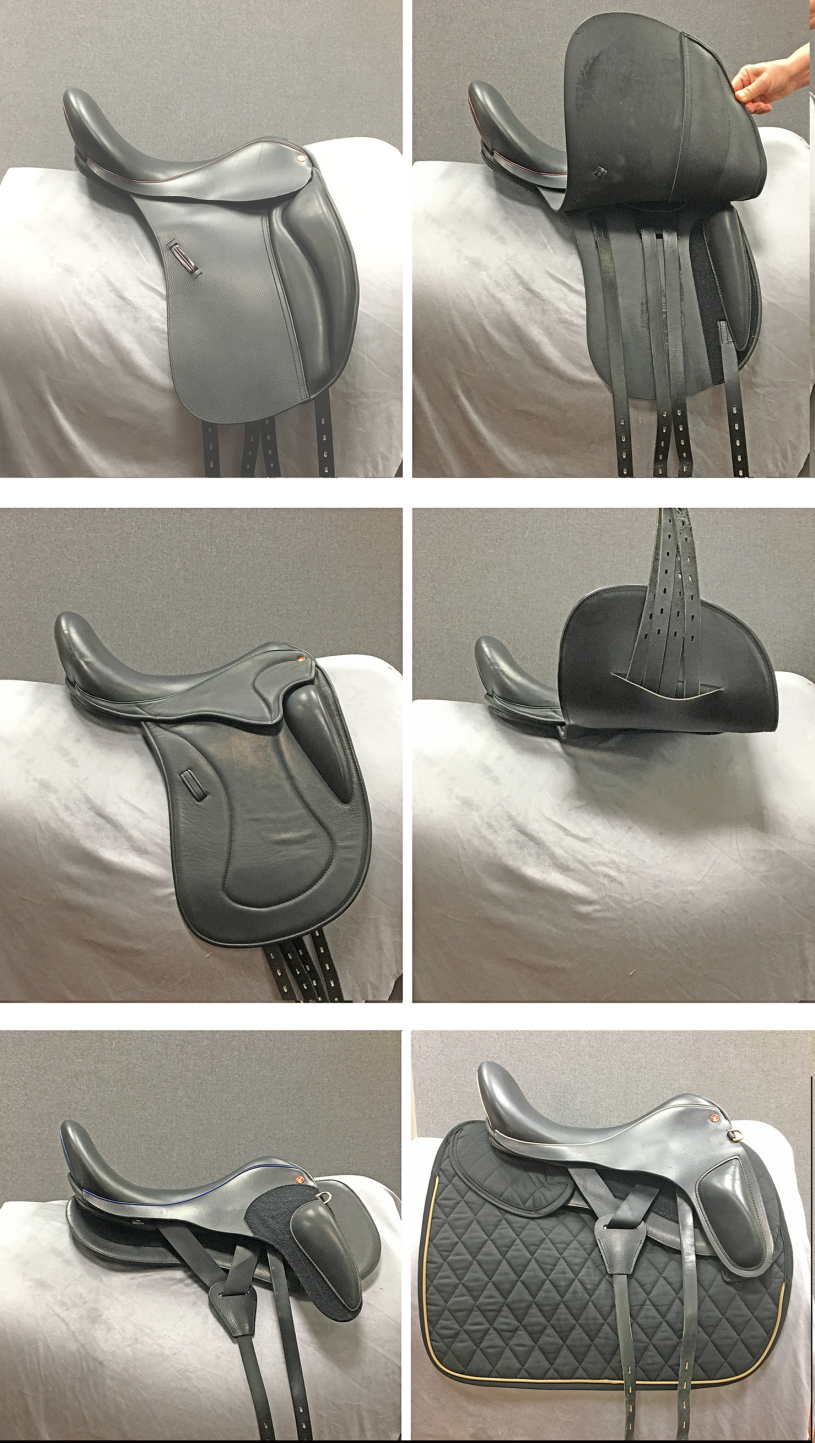
Different saddle flap configurations. Top: conventional saddle with two flaps showing the outer flap (left) and outer flap raised to show the underlying sweat flap and girth tabs (right). Middle: monoflap saddle showing the outer surface of the flap (left) and with the flap raised (right). Bottom: flapless saddle without pad (left) and with pad (right).

A conventional English saddle has as its core a rigid tree that must be sized correctly according to the width and shape of the horse’s back to avoid painful pressure points beneath the rigid parts of the tree. On the underside of the tree are the panels which are shaped to match the contours of the individual horse’s back. Above the tree is the seat of the saddle that should fit the size and shape of the rider’s pelvis, buttocks and thighs, which differ considerably from the shape of the horse’s back. The flaps extend downwards and lie between the horse’s ribcage and the rider’s leg. A conventional saddle (Fig 1) has two flaps: a sweat flap adjacent to the horse’s ribcage upon which the girth straps (billets) are located and a second flap is attached outside of the sweat flap and billets with the stirrup leathers on its outer surface. A monoflap saddle (Fig 1) has a single flap with the girth straps emerging on its underside and the stirrup leathers lying on the outside. Advantages that have been ascribed to the monoflap saddle include a reduction in saddle weight and closer contact between the rider’s leg and the horse’s ribcage. The flapless saddle (Fig 1) goes a stage further in having no flaps which places the rider’s legs even closer to the sides of the horse, from which they are separated only by a soft, cushioned saddle pad in the thigh region. The billets and stirrup leathers drape around the saddle pad that is anchored securely to the panels of the saddle and extends ventrally over the upper part of the horse’s ribcage on each side.

The rider’s center of pressure (COP) is a point representing the centroid of pressure distribution on the horse’s back. Movements of the COP occur as the rider follows the rhythmic motion of the horse’s back and the range of motion (ROM) of the COP has been used as an indication of rider stability [2]. The quality of a rider can be judged by their stability in the saddle [3] and good riders appear to be inherently motivated to improve their stability. A saddle that is a poor fit for the horse causes greater instability in the system [3]. In this study we explore whether a saddle that gives the rider a more functional leg position involving closer contact with the horse and the opportunity for greater haptic input can improve the rider’s stability.

The objective was to compare the ROM of the rider’s COP in anteroposterior (AP) and mediolateral (ML) directions at walk, trot and canter when riding with their own conventional saddle and with a prototype saddle without flaps (flapless saddle). We expect that the closer contact between the rider’s legs and the horse with the flapless saddle will result in greater stability of the rider. The experimental hypothesis is that the ROM of the rider’s COP in the AP and ML directions will be smaller with the flapless saddle compared with the conventional saddles.

## Materials and methods

The study was performed with approval of the Michigan State University Institutional Animal Care and Use Committee (protocol number 03/13-049-00). The subjects were five dressage horses (three European warmbloods, one Thoroughbred-warmblood cross, and one Lusitano; age: 9–16 years; height: 1.60 to 1.83 m) ridden by their regular rider (height: 1.73 m; weight: 61.2 kg). The same professional rider rode all the horses to reduce variability in the data, to avoid bias introduced by using multiple riders, and to ensure consistency of the motion patterns [4]. The horses were in full-time dressage training and were working between US Equestrian fourth level https://www.usef.org/compete/disciplines/dressage/dressage-tests to Fédération Equestre Internationale Grand Prix level http://inside.fei.org/fei/your-role/organisers/dressage/tests.

Each horse was ridden in its own conventional saddle, which had a tree, a sweat flap and an outer flap, and in a prototype flapless saddle that had a tree but no flaps. The flapless saddle was adjusted to fit each horse and rider combination. The horses were ridden in the flapless saddle twice during the 3 days prior to data collection to ensure that both horse and rider were familiar with its feel. The horses’ own saddles plus the rider weighed from 69.8 to 72.2 kg. The flapless saddle plus the rider weighed 70.4 kg.

Each horse/rider/saddle combination performed a pre-determined pattern of movements that included walk, sitting and rising trot, and cantering on both leads around a dressage arena in clockwise and counter-clockwise directions. Each gait was performed at slow speed with the horse taking short strides (collected) and at fast speed with the horse taking long strides (extended). Data used in this study were recorded as the horses moved in a straight line along the long side of the arena to avoid changes in the rider’s weight distribution associated with turning or bending the horse [5]. A series of 21 wooden stakes spaced 1 m apart were placed immediately behind the boards demarcating the long side of the arena to calibrate the distance measurements. A stationary video camera was set up perpendicular to, and 25 m from, the horses’ line of travel. The zoom was adjusted to encompass the 20 m length covered by the stakes. The horse’s average speed was calculated by measuring the time taken to cover an integral number of strides (over a distance of approximately 16 m) in the center of the field of view.

Pressure data were collected using a Pliance (Novel, Germany) electronic pressure mat that is partially divided into left and right halves each measuring 60 by 20 cm and having 128 embedded pressure sensors. In preparation for data collection, the pressure mat was placed on the horse’s back so the two halves lay symmetrically on each side of the dorsal midline and in a position that would accommodate the full length of the panels of both saddles. The mat did not move by more than 1 cm during the testing of any saddle so it was not considered necessary to repeat any of the tests. The position of the corners of the mat were marked on the horse’s hair coat and used as a reference to ensure that the mat was in the same position on the horse’s back for both saddles. Data from the pressure mat were transmitted via Bluetooth to a laptop computer at the side of the arena. The order in which data were collected for the two saddles was randomized between horses (randomizer.org) and determined in advance of the data collection. The first saddle was placed on the horse’s back on top of the pressure mat and the pressure mat was zeroed. The girth was tightened gradually, alternating between the left and right sides. The rider used a high mounting block to mount without weighting the stirrup and then warmed up for approximately 15 minutes until she felt the horse was ready to perform the pattern. Video and pressure data were recorded at collected and extended walk, trot and canter as the horse moved in a straight line along the long side of the arena. After completing the pattern with the first saddle the rider dismounted, the girth was loosened gradually and the saddle was removed taking care not to disturb the pressure mat. If necessary, the position of the pressure mat was adjusted to re-align it with the marks on the horse’s hair coat without removing it from the horse’s back to reduce errors [6]. The second saddle was placed on the pressure mat and the procedure was repeated.

Pressure measurements were recorded and analysed for segments of the pattern in which the horses walked, trotted and cantered along the long side of the arena. The Pliance software (version 24, Novel, Germany) was used to split the data strings into strides with five strides being analysed for collected and extended walk, trot and canter per saddle/horse combination. The path of the COP was tracked and minimal and maximal COP coordinates in the AP and ML directions were extracted on a stride-by-stride basis. From these values the AP and ML ROMs were determined by subtraction.

The outcome variables (AP ROM and ML ROM) were transformed along the ladder of powers. The most normal transformations judged from plotting and descriptive statistics were selected. The AP data were normally distributed, the ML data were negative inverse square root transformed. Comparisons between the ROM of the COP for the two saddles at the collected and extended walk, trot and canter were explored using mixed models with gait and saddle as fixed factors, including their 2-way interaction, and horse as a random effect. In addition, ROM was compared between saddles and between collected and extended variations of each gait.

## Results

**Fig 2:**
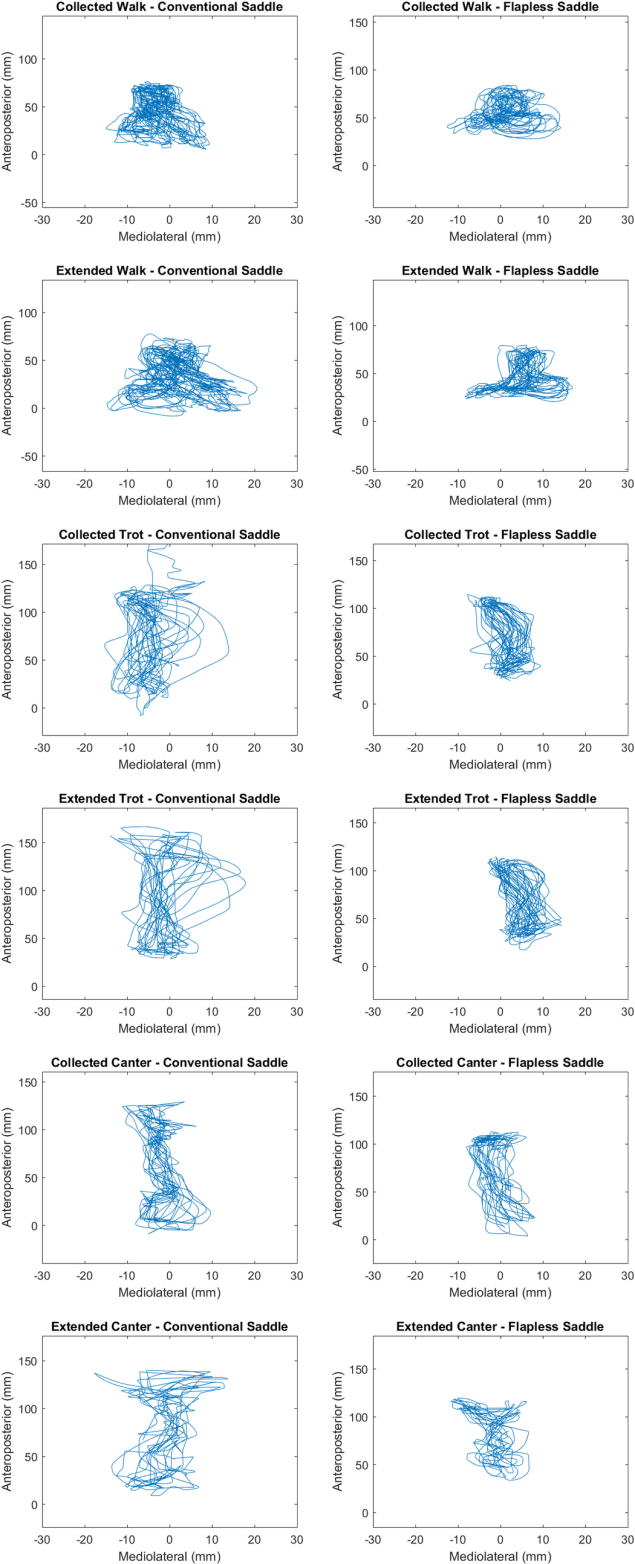
Typical center of pressure tracings for one horse-rider combination moving on a straight line. Data are shown for walk (top two rows), trot (middle two rows) and canter (bottom two rows) using a conventional saddle (left) and a flapless saddle (right).

There were no significant differences in the speed or stride length of any gait when horses were ridden in the conventional versus the flapless saddles (Table 1). The rider’s ROM in the ML direction was significantly smaller in all six gait types when riding in the flapless saddle (Table 2, Fig 2). The rider’s ROM in the AP direction was smaller in the flapless saddle at all gaits with the difference reaching statistical significance in collected trot, extended trot and extended canter (Table 2, Fig 2).

**Table 1:** Mean values and (standard deviations) of speeds and stride lengths for horses performing collected and extended walk, trot, and canter.

		Collected		Extended	
		Conventional saddle	Flapless saddle	Conventional saddle	Flapless saddle
Speed (m/s)	Walk	1.6 (0.0)	1.6 (0.1)	1.7 (0.0)	1.7 (0.0)
	Trot	3.3 (0.2)	3.3 (0.2)	4.4 (0.2)	4.4 (0.1)
	Canter	3.9 (0.2)	3.8 (0.2)	5.6 (0.3)	5.7 (0.3)
Stride length (m)	Walk	1.8 (0.1)	1.8 (0.1)	2.0 (0.1)	2.0 (0.1)
	Trot	2.6 (0.2)	2.6 (0.2)	3.3 (0.2)	3.4 (0.2)
	Canter	2.4 (0.2)	2.4 (0.2)	3.4 (0.3)	3.4 (0.2)

**Table 2:** Mean values and (standard deviations) for range of motion of the rider’s center of pressure (COP) in walk, trot and canter.

	AP ROM (mm)			ML ROM (mm)		
	Conventional saddle	Flapless saddle	P value	Conventional saddle	Flapless saddle	P value
Collected walk	56.6 (12.3)	54.7 (12.5)	0.764	19.9 (3.8)	16.8 (5.6)	0.031
Extended walk	67.4 (11.2)	64.8 (13.2)	0.675	24.9 (5.8)	20.2 (6.9)	0.014
Collected trot	102.4 (21.1)	89.3 (9.4)	0.036	26.1 (6.9)	17.9 (5.2)	<0.001
Extended trot	120.9 (21.7)	95.0 (13.9)	<0.001	50.9 (32.1)	22.8 (7.7)	<0.001
Collected canter	111.5 (19.0)	103.8 (15.2)	0.192	17.9 (5.2)	13.8 (3.6)	0.006
Extended canter	103.4 (21.2)	91.3 (16.0)	0.049	25.8 (10.4)	17.5 (4.5)	0.012

Within each gait, p-values are shown for the comparison between anteroposterior (AP) and mediolateral (ML) ranges of motion (ROM) between the conventional saddle and flapless saddle.

## Discussion

The results presented here have shown that riding in a saddle designed to provide a closer contact between the rider’s legs and the horse reduced the ML ROM of the rider’s COP in all gaits and reduced the AP ROM in collected trot, extended trot and extended canter, which partially supports the experimental hypothesis. These findings indicate that the rider is more stable from side-to-side and is better able to control lateral body movements in the face of perturbations due to gravitational, inertial and ground reaction forces (GRF) generated during locomotion.

The rider’s position in the saddle is the basis for effective communication between the rider and the horse [4]. Ideally, a dressage rider sits with the ear, shoulder, hip and heel in vertical alignment when viewed in the sagittal plane. In the frontal plane, the head, trunk and pelvis should be vertically aligned and centered over the midline of the horse with the thighs draped lightly around the horse’s ribcage and the toes pointing forward [7]. This position should be maintained, as far as possible, when the horse is in motion.

The rhythmic motion of the horse’s back involves translational and rotational movements that have a characteristic pattern in each gait [8,9] and that vary with speed within each gait [10]. Therefore, it was considered important to control speed within each gait type in order to make valid comparisons between saddles. The movements of the horse’s back are responsible for predictable perturbations of the rider’s position that result in the typical excursion pattern of the rider’s COM in each gait [11,12,13,14]. The ability to control body movements in the face of these perturbations is a characteristic of an experienced rider [15]. The horse’s trunk pitches from front to back in the sagittal plane and rolls from side to side in the frontal plane to varying degrees in different gaits. As a general principle, the rider’s pelvis pitches in the opposite direction to the saddle and rolls in the same direction as the saddle though the movements are somewhat out-of-phase in trot and canter [15–19]. The direction of rotation of the rider’s trunk differs between individuals and is less consistent than pelvic rotations [17].

The coupling between horse and rider via the saddle affects not only the movements of the rider’s COP but also the movements of the entire horse-rider system [20]. Peham et al. [4] showed that sitting trot has a larger AP ROM of the COP than rising trot or a two-point seat. However, dressage riders do not have the option to use the more stable rising or two-point positions in competition. The smaller AP ROM in trot with the flapless saddle is likely to be beneficial because stability of the rider’s seat is necessary for effective communication between the rider and horse. Different saddles can affect the motion patterns of both horse [3] and rider [21]. The flapless saddle, which offers closer contact between the rider’s legs and the horse, and the associated reduction in ROM of the rider’s COP indicates a more tightly controlled system. This could be a result of changes in rider kinematics resulting in favourable muscular and biomechanical adaptations or it could be associated with improved haptic or tactile information as a result of closer contact of the rider’s legs with the horse [22].

An experienced rider anticipates the rhythmic movements of the horse’s back and uses a feed-forward mechanism to move in phase with the horse’s body [23] with only small temporal offsets between the movements of horse and rider [24]. Unexpected perturbations arising as a result of unpredictable movements by the horse are countered using a feedback mechanism based on proprioceptive input. The rider’s ability to stabilize the body in the face of these perturbations is highly dependent on controlling the lumbopelvic-hip complex, which includes the lumbar spine, the pelvis, the hip joints and the active and passive soft tissues responsible for causing and controlling their movements [25].

The lines of action of the abdominal and epaxial musculature are such that they provide greater trunk stability in the AP than in the ML direction, which is beneficial for a rider because the largest accelerations of the horse’s body are due to the effects of the braking and propulsive longitudinal GRFs. This may also explain the strong in-phase coupling between rider and horse in the AP direction, especially in the trot in which the braking and longitudinal forces occur synchronously in the fore and hind limbs. Co-activation of the antagonistic core muscles makes the spine system more robust to external perturbations [26] but the degree of co-activation should be appropriate to the task at hand [27].

Synchrony between the horse and rider’s motion is highest in canter due to the acceleration and deceleration of the different limbs being out of phase so the accelerative and decelerative effects on the rider are less pronounced [24]. However, canter is characterized by having a large pitching rotation in each stride with a correspondingly large range of pelvic rotation in the rider. The rider follows the motion of the saddle with a posterior pelvic rotation, which is likely to account for much of the AP motion of the COM. Thus, although the AP COP has a similar ROM in trot and canter, the underlying mechanisms are somewhat different.

In the frontal plane, ML stabilization of the trunk keeps the rider centered over the horse. It is a characteristic of experienced riders that they maintain their pelvis in a position close to the horse’s midline [19], which is evidence of an advanced level of postural control in the riding position. The most dramatic effect of the flapless saddle was reduced ML motion of the COP. The COP shifts laterally as a consequence of lateral translation or roll rotation of the pelvis and/or trunk segments, with the COP moving toward the more heavily loaded side. Reduction of the lateral ROM of the COP implies better control of lateral translations and rotations of the pelvis and trunk. Since the knee joint acts as a hinge in flexion-extension, the hip joint must be somewhat flexed, abducted, and/or externally rotated to allow the rider’s thigh and calf to conform to the convexity of the horse’s ribcage. One of the benefits of the flapless saddle is that by removing two layers of leather on each side, it narrows the distance between the rider’s thighs and the distance between the rider’s knees, which requires less flexion/abduction/external rotation of the hip joint.

Since the core musculature is less effective in stabilizing the trunk in the ML direction there is a weaker coupling between rider and horse in this direction [19]. The greater difficulty in maintaining ML stability was illustrated in a study that compared ROM of the COP at the walk in able-bodied riders and riders with cerebral palsy. Differences between the two groups were most evident in the ML direction. Even at walk, riders with cerebral palsy had a significantly larger ML ROM than able-bodied riders due to their reduced ability to control lateral trunk movements and resist ML perturbations from the horse [2]. In the study reported here, the gaits showing the greatest increase in ML stabilization with the flapless saddle also had large improvements in AP stabilization.

Limitations of the study are the small number of horses and the fact that the rider could not be blinded to which saddle she was riding in. Future studies should evaluate a larger number of riders of diverse morphological types and should select horses that vary in conformation, particularly in ribcage dimensions and proportions. Comparison with a monoflap saddle might also be interesting.

## Conclusions

In conclusion, the use of a flapless saddle that allowed closer contact of the rider’s legs with the sides of the horse’s ribcage significantly improved rider stability, particularly in the ML direction. In the AP direction the rider was better able to control movements of the COP, most notably in the trot in which large longitudinal braking and propulsive forces are applied. The results suggest that further investigations of horse conformation, rider morphology, and their interaction with different types of equipment are warranted.
